# Designing a Virtual Reality Café to Treat Eating Disorders: A Thematic Analysis of Stakeholder Viewpoints

**DOI:** 10.1192/bjo.2024.244

**Published:** 2024-08-01

**Authors:** Lydia Shackshaft, Laura Chapman, Lucy Biddle, Lucy Yardley, Helen Bould

**Affiliations:** 1 Centre for Academic Mental Health, Population Health Sciences, Bristol Medical School, University of Bristol, Bristol, United Kingdom; 2 University Hospitals Bristol and Weston NHS Foundation Trust, Bristol, United Kingdom; 3 School of Psychological Science, University of Bristol, Bristol, United Kingdom; 4 Bristol Medical School, University of Bristol, Bristol, United Kingdom

## Abstract

**Aims:**

Eating disorders (ED) have significant physical and psychosocial impacts, and the highest mortality rates of any psychiatric illness. About a third of patients with Anorexia Nervosa or Bulimia Nervosa do not recover and develop persistent ED. Development of novel treatments is a priority to prevent adverse effects on young people's physical, relational and educational development. Virtual reality (VR) has shown promising efficacy as an innovative mental health treatment, and has potential therapeutic value within ED. People with lived experience (PWLE) and clinicians have demonstrated enthusiasm for a VR café intervention to practice social and food-related challenges. A VR café would enable gradual exposure to challenges in a protected environment, aiming to support people with ED to return to real-life cafés and social eating. This study aims to explore the opinions of key stakeholders to help inform the development of a VR café scenario as an adjunctive treatment for ED.

**Methods:**

We conducted semi-structured focus groups and 1:1 interviews with PWLE aged 14–25 years (n = 15), parents/carers (n = 4), and clinicians (n = 6). Participants were recruited via social media, advertisement via ED charities, posters in public places, and snowballing. Following completion of an online screening survey, eligible individuals were invited to participate using purposive sampling to ensure diversity of ages, ethnicities, genders, ED diagnoses, and health professional roles. Data were analysed thematically.

**Results:**

Preliminary analysis indicates that PWLE, parents/carers and clinicians expressed mostly positive opinions regarding a VR café adjunctive treatment. Expressed concerns related to themes of intervention efficacy, translation of learnt skills to real life, and use of VR technology. Most participants agreed a VR café intervention should be a repeated experience (many suggested graded exposure), realistic, and maximally individualised. All stakeholder groups identified a similar range of challenges to experience within a VR café, with themes including choosing food, other people, eating socially or alone, and the café environment. Differences in specific aspects of the scenario that might make challenges harder or easier reflected the unique experiences of individual participants.

**Conclusion:**

These findings build upon previous research demonstrating support from PWLE, parents/carers, and clinicians for the development of a VR café adjunctive treatment for ED. Themes identified are largely consistent across stakeholder groups and relate to the design of a VR café scenario and its implementation as a treatment. This analysis enables the perspectives of key stakeholders to be incorporated into the design of a novel VR café intervention to optimise efficacy and acceptability.

